# Optimal resource allocation method for energy harvesting based underlay Cognitive Radio networks

**DOI:** 10.1371/journal.pone.0279886

**Published:** 2023-01-05

**Authors:** Jianbin Liao, Hongliang Yu, Weibin Jiang, Ruiquan Lin, Jun Wang

**Affiliations:** 1 Marine engineering college, Dalian Maritime University, Dalian, China; 2 Fujian Province Key Laboratory of Ship and Ocean Engineering, Xiamen, Fujian, China; 3 College of Electrical Engineering and Automation, Fuzhou University, Fuzhou, China; Norfolk State University, UNITED STATES

## Abstract

This paper proposes an optimal resource allocation method. The method is to maximize the Energy Efficiency (EE) for an Energy Harvesting (EH) enabled underlay Cognitive Radio (CR) network. First, we assumed the Secondary Users (SUs) can harvest energy from the surrounding Radio Frequency (RF) signals. Then, we modelled the EE maximisation problem as a joint time and power optimization model. Next, the optimal EH time allocation factor can be calculated. After that the optimal power allocation strategy can be obtain by the fractional programming and Lagrange multiplier method. Finally simulation results show that the proposed iterative method can be better performance advantages compared with the exhaustive method and genetic algorithm. And the EE of this system model is significantly improved compared to the EE model without considering EH.

## 1 Introduction

With the continuous development of wireless communication technology, the limited spectrum resources can hardly meet the needs of wireless users. Cognitive Radio (CR) is a wireless communication paradigm in which the unlicensed users or Secondary Users (SUs) who have no spectrum licenses can opportunistically access and use the unused spectrum of licensed users or Primary Users (PUs) who have spectrum licenses without causing interference to the PUs [1, 2]. In a typical CR system, SUs first perform spectrum sensing to find the unused bands and then adjust their transmission parameters such as coding schemes, modulation schemes, and transmitting power to access the unused bands. There are three types of spectrum sharing techniques in CR systems: interweave, underlay and overlay [3]. In interweave CR systems, SUs can only use the unused bands of PUs and in underlay CR systems, SUs can use the bands below certain power limits without causing interference to PUs. In overlay CR systems, SUs actively help in PUs’ data transmission. In overlay and interweave CR systems, SUs can access the spectrum licensed to PU only when the spectrum is not occupied by PU. Spectrum sensing establishes the spectrum occupation status of the PU’s. In the underlying CR system, SUs do not need to perform spectrum sensing to determine the spectrum-occupation status of the PU, and they are permitted to access the PU’s spectrum even when the PU is active.

As wireless communication technology replaces wired technology in a large area and the number of cognitive users increases rapidly, the idle spectrum under the overlay spectrum sharing mode becomes more and more crowded. Because of this practical problem that will be faced in the future, it becomes very necessary to study the underlay spectrum resource sharing problem in which cognitive users and primary users use the same channel at the same time. In this paper, we only considered an underlay CR network. The SUs can share channels with the PU for data transmission without interfering with the PU’s data transmission. In CR, PUs are the authorized users and have the priority to access spectrum bands. SUs adjust transmission power according to the perceived spectrum occupancy information and radio environment information to avoid harmful interference to PUs when SUs try to access the authorized channels.

In a CR network, a proper resource allocation method can achieve a high spectrum utilization rate and energy utilization rate. [4] proposed a joint EE and transmission time allocation method for the CR network. The method can obtain optimal sensing time and transmission time simultaneously. The method enables the network to obtain optimal sensing time and transmission time simultaneously. The detection probability and reoccupation probability are set in advance to protect the PUs. [5] considerd the interference power limit and proposed the optimal power allocation strategy to maximize the SUs’ EE over fading channels. [6] proposed resource allocation methods that adopt spectrum sharing combined with soft-sensing information, adaptive sensing thresholds, and adaptive power to maximum the EE. However, these papers only propose the EE maximization problem based on cognitive wireless networks without EH.

Meanwhile, more and more services and applications would be performed by communication devices today. The energy consumption of a communication network increases dramatically. Therefore, EH technology is proposed to be applied to CR networks by some researchers [7–9], which may solve the shortage of energy and partially realize green communication. EH is a technology that supports continuous power supply in energy-constrained communication systems. Because of its promise, it has received extensive attention in recent years. The EH efficiency is very low, but it is still suitable for low-power communication equipment. The source of RF energy includes dedicated RF source, ambient RF energy and self-power, among which dedicated RF energy has properties of continuity and stability and is more practical compared with the others [10, 11]. In [12], a RF EH-CR sensor network is proposed, which can utilize the strong signals over the occupied licensed channels for EH.

The integration of CR and EH has become the focus of green communication research. [13] consider a multi-user MIMO SWIPT network having a multi-antenna BS serving multiple multi-antenna information receivers while ensuring a minimum harvested power at multiple multi-antenna energy receivers. However, [13] mainly proposed a power optimization method, not a joint time and power optimization method. The work in [14] studied how to make full use of multi-dimensional resources and maximize the sum throughput of the Internet of Things (IoT) devices in a SWIPT-enabled cognitive IoT network. However, [14] is for an interweave CR network and its goal is to maximize the sum throughput of the network and not the energy efficiency of the network. In [15], the maximum EE of SU was achieved by optimizing time and power under energy causality constraint and minimum throughput constraints for interweave CR networks. The work in [16] proposed an energy consumption minimization method for EH-CR network during the spectrum sensing phase of PU localization. The method can enhance the network lifetime and the EE of SU without compromising localization accuracy. In [17], a joint sensing and power allocation method for EH enabled CR network was discussed, in which SU can harvest energy during spectrum sensing. But the aim of [17] was also to maximize the sum throughput not the energy efficiency. [18] proposed a cooperative secrecy transmission mechanism that can take advantage of the transmitter signal of the primary user (PU) as a dedicated radio frequency (RF) source with decode and forward (DF) UAV selection and energy harvesting (EH) under a cognitive network. By using the function analysis method, the closed expressions of the system secrecy outage probability (SOP) and the probability of non-zero secrecy capacity were calculated and derived accurately. [19] investigated a novel transmission protocol and analyze the network’s performance. Considering the non-linear energy harvesting (EH) mechanism at power-constrained nodes and direct and cooperative phase transmissions, the outage probability (OP) and block error rate (BLER) performances are evaluated for Rayleigh distributed fading channels. The analytical results are validated through Monte-Carlo simulations. [20] was aimed at practical scenarios of small cell networks by jointly evaluating capable of interference management and EH. It was proposed transmission approaches including full duplex (FD) and bi-directional transmission to improve the main performance system metrics such as outage probability and throughput. Three useful schemes are explored by considering EH and inter-cell interference. In general, the above mentioned do not consider a joint time and power optimization of EH based cognitive wireless networks in underlay mode to maximize the EE.

In this paper, it is proposed an optimized resource allocation method aiming at maximizing the EE for an underlay EH-CR network. The main contributions can be summarized as follows:

- It modelled the EE maximisation problem as a joint time and power optimization model. Under the constraints of interference power and EH, the optimal time and power allocation is derived to maximise the EE of cognitive wireless networks, which will improve the utilisation rate of the spectrum resource.
- The optimal EH time allocation factor is calculated by optimizing the EH constraint conditions. The non-convex problem is transformed into a simple convex problem by fractional programming. Based on the optimal solution, it is proposed an iterative method to find the optimal power allocation strategy.
- It is verified by simulation that the proposed iterative method has good convergence, and the proposed iterative method has better performance advantages when compared with the exhaustive method and genetic algorithm. Compared with the traditional network without EH, the EE of the proposed method is improved.

## 2 System model

**Fig 1:**
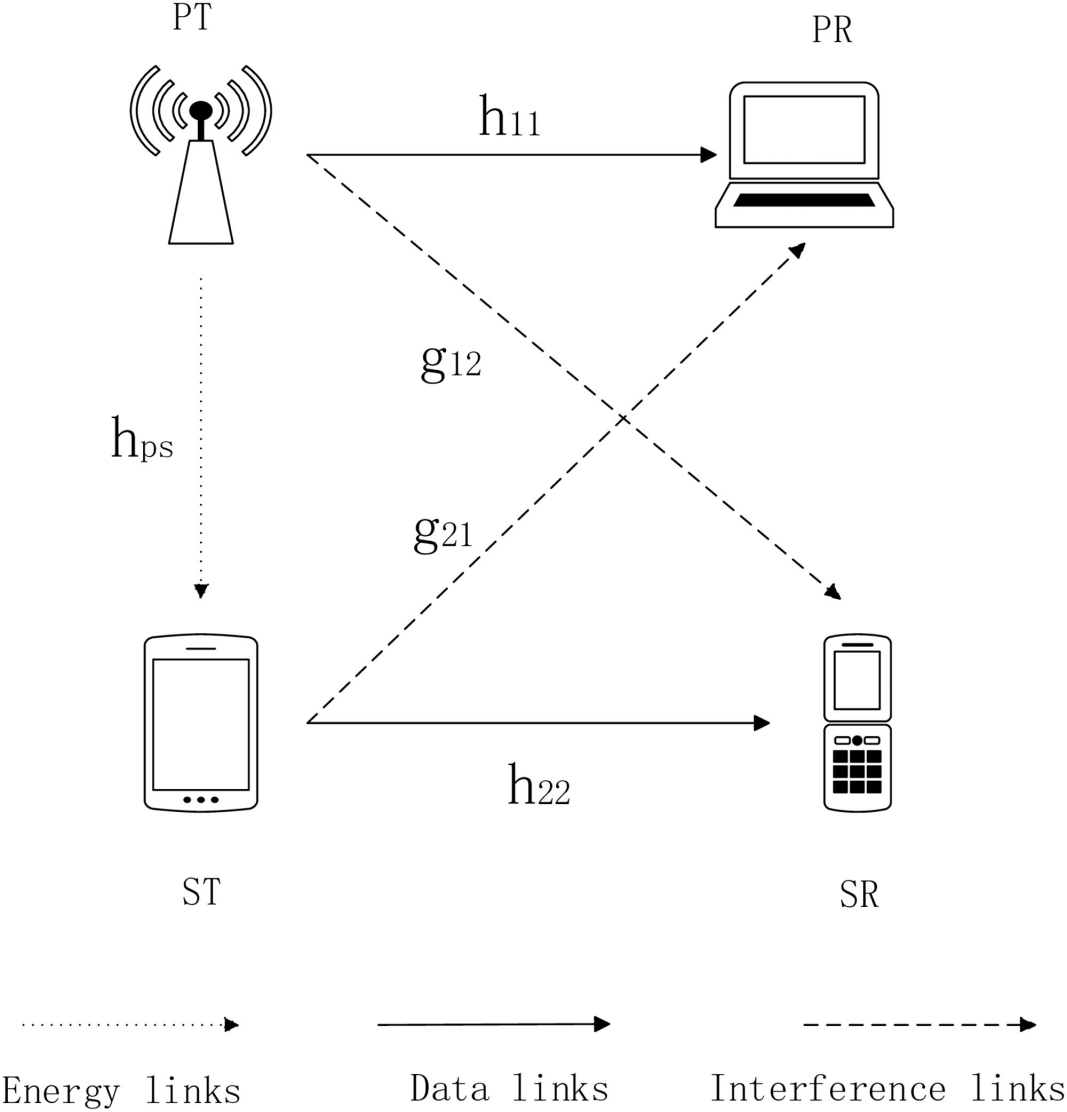
Energy harvesting cognitive radio system model.

As shown in Fig 1, this paper considers a cognitive wireless network in underlay mode with one PU Transmitter (PT)—PU Receiver (PR) pair and one SU Transmitter (ST)—SU Receiver (SR) pair. The SU uses the authorized channel of the PU to transmit data without interfering with the transmission of the PU. In order to ensure a stable and adequate supply of electricity, many dedicated RF sources are deployed for strong energy emission. And each energy harvester in a ST must be equipped with a power conversion circuit that can extra DC power from the received electromagnetic waves [21–23]. It is also assumed that the ST is equipped with two batteries. One is a rechargeable battery to store the harvested RF energy and the other is a standby non-rechargeable battery. When the harvested energy is consumed up, the ST begins to consume the energy of the non-rechargeable battery. The maximum capacity of a rechargeable battery is *E*_*max*_.

The channel is assumed to be Rayleigh block fading during data transmission. It is considered that a cognitive wireless network has *N* channels. Assuming that the channel gains on channel *i* of PT-PR, ST-SR, PT-ST, PT-SR and ST-PR link are represented as *h*_*i*,11_, *h*_*i*,22_, *g*_*i*,12_ and *g*_*i*,21_. The background noise is an Additive White Gaussian Noise (AWGN) with a mean of 0 and a variance of *σ*^2^.

**Fig 2:**
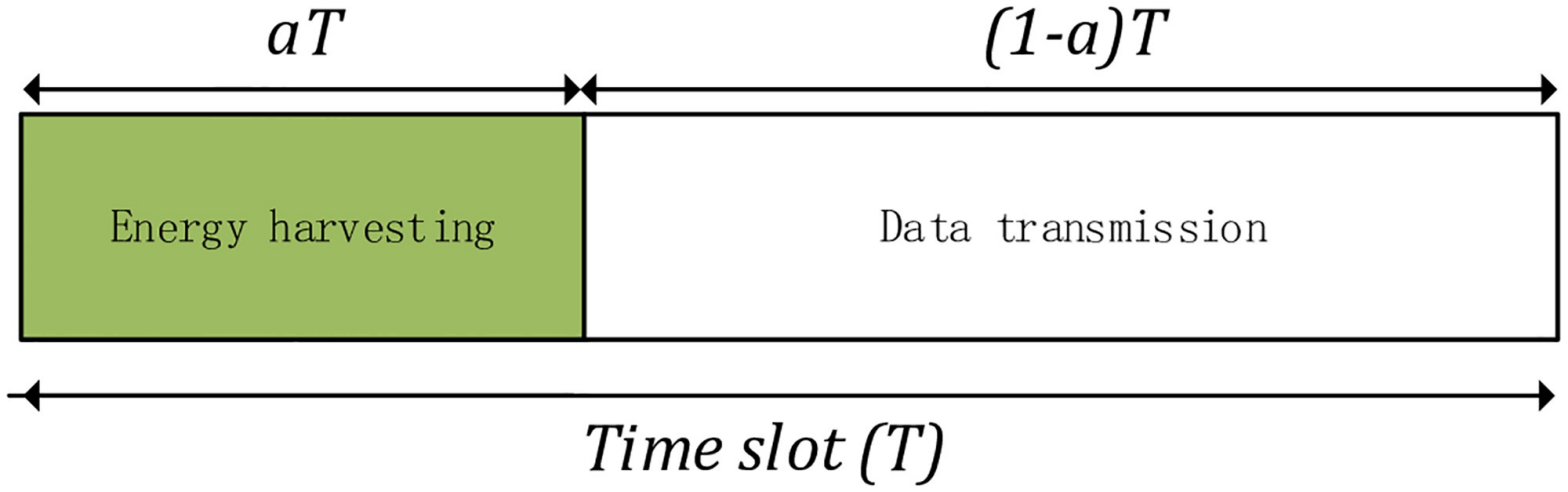
Time slot structure.

At the beginning of the time slot, the ST has cognitive ability and can sense Channel State Information (CSI) from a common control channel. The energy consumed by this sensing process is so small that it can be ignored. Therefore, the ST knows the channel power gain before data transmission. The channel has two channel states: on one hand, the PU is occupying the channel to transmit data, on the other hand, the PU is not occupying the channel to transmit data. When the channel is busy, the transmitting power of the SU is less than the interference power. Therefore, in the case of busy state as shown in Fig 2: EH and data transmission. The duration of the EH phase is *αT* and the duration of the data transmission phase is (1 − *α*)*T*, where *α* is the EH time allocation factor. The time the rechargeable battery needs to be charged is ignored here.

Using the analysis method of [24], the *E*_1_ to represent the energy consumed by data transmission and the circuit, and *E*_2_ to represent the energy collected in the EH, so we can get the net consumed energy is *E*_3_ = *E*_1_ − *E*_2_.

In the first phase of EH, we assumed that the harvested energy could at least meet the consumption of its own circuit and all the harvested energy is used up in current time slot [25]. The energy harvested at this stage is:

$$\begin{array}{c} E_{h}=\varphi P_{T}h_{ps}\alpha T \end{array}$$ (1)

where *φ* is the energy conversion efficiency, *P*_*T*_ is the transmitted power of PT, *h*_*ps*_ is the EH gain.

In the second phase of data transmission, the ST shares the spectrum resources of the PT in the underlay cognitive wireless working mode. In order to ensure the communication quality of the PU is not disturbed, the transmission power should be less than the interference power *P*_*I*_. The interference power of the system is:

$$\begin{array}{c} \sum_{i=1}^{N}P_{i,s}g_{i,12}\leq P_{I} \end{array}$$ (2)

Therefore, the throughput achieved at the SU is:

$$\begin{array}{c} R\left(\alpha ,P_{i,s}\right)=\sum_{i=1}^{N}\left(1-\alpha \right)Tlog_{2}\left(1+\frac{P_{i,s}h_{i,22}}{P_{T}g_{i,12}+\sigma^{2}}\right) \end{array}$$ (3)

where *P*_*i*, *s*_ is the transmitted power of the ST to *i*-th channel.

## 3 The proposed method

In this section, it is investigated the optimal transmission strategy for the SU to maximize the EE while satisfying the EH constraints under the transmission power constraint of the SU and the interference power constraint of the PR link. The EE is defined as the ratio of the throughput of the SU to the energy consumed by the non-rechargeable battery. The EE equation of the SU is constructed as:

$$\begin{array}{c} P1\;:\;\max_{\alpha ,P_{i,s}}\;EE=\frac{\sum_{i=1}^{N}\left(1-\alpha \right)Tlog_{2}\left(1+\frac{P_{i,s}h_{i,22}}{P_{T}g_{i,12}+\sigma^{2}}\right)}{P_{c}T+\sum_{i=1}^{N}P_{i,s}\left(1-\alpha \right)T-E_{h}} \end{array}$$ (4)

where *P*_*c*_ is the circuit power consumption of the ST.

### 3.1 Optimization of energy harvesting time

In the EH phase, the harvested battery energy meets its own circuit consumption, and the existing energy constraints are:

$$\begin{array}{c} \varphi P_{T}h_{ps}\alpha T\geq P_{c}T \end{array}$$ (5)

From the energy constraints it can be gotten:

$$\begin{array}{c} \alpha \geq \frac{P_{c}}{\varphi P_{T}h_{ps}} \end{array}$$ (6)

During the whole time slot, the energy harvested by the battery does not exceed the total energy consumed during the data transmission. The energy constraints are as:

$$\begin{array}{c} \varphi P_{T}h_{ps}\alpha T\leq P_{c}T+\sum_{i=1}^{N}P_{i,s}\left(1-\alpha \right)T \end{array}$$ (7)

From the above energy constraints it can be gotten:

$$\begin{array}{c} \alpha \leq \frac{\sum_{i=1}^{N}P_{i,s}+P_{c}}{\varphi P_{T}h_{ps}+\sum_{i=1}^{N}P_{i,s}} \end{array}$$ (8)

Taking the first derivative of P1 with respect to *α*_*i*_, then it can be gotten:

$$\begin{array}{c} \frac{\partial EE}{\partial \alpha }=\frac{\sum_{i=1}^{N}bT^{2}log_{2}\left(1+\frac{P_{i,s}h_{i,22}}{P_{T}g_{i,12}+\sigma^{2}}\right)}{{\left(P_{c}T+\sum_{i=1}^{N}P_{i,s}\left(1-\alpha \right)T-E_{h}\right)}^{2}} \end{array}$$ (9)

where *b* = *φP*_*T*_ *h*_*ps*_ − *P*_*c*_. From Eq (5), it can be gotten $\sum_{i=1}^{N}bT^{2}log_{2}\left(1+\frac{P_{i,s}h_{i,22}}{P_{T}g_{i,12}+\sigma^{2}}\right)\geq 0$, so the EE function is increasing with respect to the EH time allocation factor *α*_*i*_. Combined Eq (6) and Eq (8), the EH time allocation factor can be obtained in the range of:

$$\begin{array}{c} \frac{P_{c}}{\varphi P_{T}h_{ps}}\leq \alpha \leq \frac{\sum_{i=1}^{N}P_{i,s}+P_{c}}{\varphi P_{T}h_{ps}+\sum_{i=1}^{N}P_{i,s}} \end{array}$$ (10)

### 3.2 Energy-efficient optimal power allocation method

The problem of maximizing the EE of the ST transmitting power *P*_*i*, *s*_ can be formulated as follows:

$$\begin{array}{c} P2\;:\;\max_{P_{i,s}}EE=\frac{\sum_{i=1}^{N}\left(1-\alpha \right)Tlog_{2}\left(1+\frac{P_{i,s}h_{i,22}}{P_{T}g_{i,12}+\sigma^{2}}\right)}{P_{c}T+\sum_{i=1}^{N}P_{i,s}\left(1-\alpha \right)T-E_{h}} \end{array}$$ (11)

$$\begin{array}{c} s.t.\{\begin{array}{c} C_{1}\;:\;\sum_{i=1}^{N}P_{i,s}g_{i,12}\leq P_{I}\\ C_{2}\;:\;E_{h}\leq P_{c}T+\sum_{i=1}^{N}P_{i,s}\left(1-\alpha \right)T\\ C_{3}\;:\;\sum_{i=1}^{N}P_{i,s}\geq 0\\ C_{4}\;:\;\sum_{i=1}^{N}P_{i,s}\leq P_{max}\leq P_{I} \end{array} \end{array}$$ (12)

In order to obtain the maximum value of *P*_*i*, *s*_, the fractional programming problem P2 can be equivalent to Dinkelbach’s method [26]:

$$\begin{array}{c} \begin{array}{cc} P3\;:\;\max_{P_{i,s}} & f\left(\eta \right)=\sum_{i=1}^{N}\left(1-\alpha \right)Tlog_{2}\left(1+\frac{P_{i,s}h_{i,22}}{P_{T}g_{i,12}+\sigma^{2}}\right)\\ & -\eta (P_{c}T+\sum_{i=1}^{N}P_{i,s}\left(1-\alpha \right)T-E_{h}) \end{array} \end{array}$$ (13)

$$\begin{array}{c} s.t.\{\begin{array}{c} C_{1}\;:\;\sum_{i=1}^{N}P_{i,s}g_{i,12}\leq P_{I}\\ C_{2}\;:\;E_{h}\leq P_{c}T+\sum_{i=1}^{N}P_{i,s}\left(1-\alpha \right)T\\ C_{3}\;:\;\sum_{i=1}^{N}P_{i,s}\geq 0\\ C_{4}\;:\;\sum_{i=1}^{N}P_{i,s}\leq P_{max}\leq P_{I} \end{array} \end{array}$$ (14)

where *η* is a non-negative parameter. When *f* (η^*^) = 0, the energy efficiency function reaches its maximum value. An auxiliary variable *E*_*i*, *s*_ = *P*_*i*, *s*_ (1 − *α*)*T* is introduced into question P3 to represent the energy consumed by SU in the transmission stage. The problem of P3 optimization can be rewritten as:

$$\begin{array}{c} \begin{array}{cc} P4\;:\;\max_{p} & f\left(\eta \right)=\sum_{i=1}^{N}\left(1-\alpha \right)T\\ & \times log_{2}\left(1+\frac{E_{i,s}h_{i,22}}{\left(P_{T}g_{i,12}+\sigma^{2}\right)\left(1-\alpha \right)T}\right)\\ & -\eta (P_{c}T+\sum_{i=1}^{N}E_{i,s}-E_{h}) \end{array} \end{array}$$ (15)

$$\begin{array}{c} s.t.\{\begin{array}{c} C_{1}\;:\;\sum_{i=1}^{N}E_{i,s}g_{i,12}\leq P_{I}\left(1-\alpha \right)T\\ C_{2}\;:\;\varphi P_{T}h_{ps}\alpha T\leq P_{c}T+\sum_{i=1}^{N}E_{i,s} \end{array} \end{array}$$ (16)

where $p=0\leq \sum_{i=1}^{N}P_{i,s}\leq P_{max}\leq P_{I}$.

**Lemma 1**. For problem *max*{*N*(*x*)/*D*(*x*)|*x* ∈ *s*}, where *N*(*x*) and *D*(*x*) are both positive continuous functions. The following properties exist: if and only if Eq (17) is satisfied, Eq (18) holds true [27].

$$\begin{array}{c} F\left(\eta_{0}\right)=F\left(\eta_{0},x_{0}\right)=max\left\{N\left(x\right)-\eta_{0}D\left(x\right)|x\in s\right\}=0 \end{array}$$ (17)

$$\begin{array}{c} \eta_{0}=N\left(x\right)/D\left(x\right)=max\left\{N\left(x\right)/D\left(x\right)|x\in s\right\} \end{array}$$ (18)

A concrete proof of **lemma 1** is in [27]. According to **Lemma 1**, the optimal solution of P4 is the optimal solution of P2. At this point, the objective function of P4 is a joint concave function and all the constraints are convex sets. The transformation problem P4 is a standard convex optimization problem. Lagrange multiplier method is used to solve P4 as follows:

$$\begin{array}{c} \begin{array}{cc} & L\left(E_{i,s},\lambda ,\upsilon \right)=\sum_{i=1}^{N}\left(1-\alpha \right)T\\ & \times log_{2}\left(1+\frac{E_{i,s}h_{i,22}}{\left(1-\alpha \right)T\left(P_{T}g_{i,12}+\sigma^{2}\right)}\right)\\ & -\eta (P_{c}T+\sum_{i=1}^{N}E_{i,s}-E_{h})\\ & -\lambda (\sum_{i=1}^{N}E_{i,s}g_{i,12}-P_{I}\left(1-\alpha \right)T)\\ & -\upsilon (\varphi P_{T}h_{ps}\alpha T-P_{c}T-\sum_{i=1}^{N}E_{i,s}) \end{array} \end{array}$$ (19)

where λ and *υ* are the non-negative dual variables. Based on the Karush-Kuhn-Tucker (KKT) conditions $\frac{\partial L}{\partial E_{i,s}}=0$, namely:

$$\begin{array}{c} \begin{array}{cc} \frac{\partial L}{\partial E_{i,s}}= & \frac{h_{i,22}\left(1-\alpha \right)T}{ln2[\left(P_{T}g_{i,12}+\sigma^{2}\right)\left(1-\alpha \right)T+E_{i,s}h_{i,22}]}\\ & -\eta -\lambda g_{12}+\upsilon =0 \end{array} \end{array}$$ (20)

From Eq (20), it can be gotten the solution of transmitting power *P*_*i*, *s*_ of the ST as:

$$\begin{array}{c} P_{i,s}=\frac{E_{i,s}}{(1-\alpha )T}={\left[\frac{1}{(\eta +\lambda g_{i,12}-\upsilon )ln2}-\frac{P_{T}g_{i,12}+\sigma^{2}}{h_{i,22}}\right]}^{+} \end{array}$$ (21)

In order to calculate the transmission power *P*_*i*, *s*_, the subgradient method can be used to update the Lagrange multiplier {λ, *υ*}. The obtained iterative formula is:

$$\left\{ \begin{array}{c} \lambda^{\left(k+1\right)}={\left[\lambda^{\left(k\right)}-s\left(\sum_{i=1}^{N}E_{i,s}g_{i,12}-P_{I}\left(1-\alpha \right)T\right)\right]}^{+}\\ \begin{array}{cc} \upsilon^{\left(k+1\right)}= & {\left[\upsilon^{\left(k\right)}-s\left(\varphi P_{T}h_{ps}\alpha T-P_{c}T-\sum_{i=1}^{N}E_{i,s}\right)\right]}^{+} \end{array} \end{array}\right.$$ (22)

where *k* and *s* represent the number and step of iteration respectively. Due to the concavity of problem Eq (15), the iterative optimization between *P*_*s*_ and {λ, *υ*} will finally converge to the optimal solution of Eq (15) [7]. Considering the transmitted power and energy constraints of the ST, it can be concluded that the optimal power allocation in energy-efficient transmission is:

$$\begin{array}{c} P_{i,s}^{*}=min\left\{P_{i,s},P_{max}\right\} \end{array}$$ (23)

It can be seen that P3 can effectively solve the given value *η* through the energy-efficient optimal power allocation strategy in Eq (23). To find the optimal η^*^ in P3, it is resorted to the Dinkelbach’s method which is very suitable to handle the fractional programming and proved to have a superlinear convergence rate [7]. The optimal power allocation method based on EH and energy-efficient transmission, denoted by Algorithm 1.

**Algorithm 1** The energy-efficient optimal power allocation method for P2

**Input**: the error tolerances *ξ*_1_, *ξ*_2_, *ξ*_3_ > 0, the step size *s* > 0, the maximum iteration number *N*;

**Output**: decomposed modes $\left\{P_{i,s}^{*},\eta^{*}\right\}$

1: Initialization: energy efficiency $\eta_{EE}^{n}$ and dual variables λ^(0)^ = λ_0_, *υ*^(0)^ = *υ*_0_, the iteration index *n* = 0;

2: calculate *P*_*s*_ using Eq (21);

3: update λ and *υ* using the subgradient method as follows;

4: k = 0

5: **repeat**

6:  $\lambda^{\left(k+1\right)}={\left[\lambda^{\left(k\right)}-s\left(\sum_{i=1}^{N}E_{i,s}g_{i,12}-P_{I}\left(1-\alpha \right)T\right)\right]}^{+}$;

7:  $\upsilon^{\left(k+1\right)}={\left[\upsilon^{\left(k\right)}-s\left(\varphi P_{T}h_{ps}\alpha T-P_{c}T-\sum_{i=1}^{N}E_{i,s}\right)\right]}^{+}$;

8:  k = k+1;

9: **until** $|\lambda^{\left(k\right)}-\left(\sum_{i=1}^{N}E_{i,s}g_{i,12}-P_{I}\left(1-\alpha \right)T\right)|\leq \xi_{1}$ and $|\upsilon^{\left(k\right)}-\left(\varphi P_{T}h_{ps}\alpha T-P_{c}T-\sum_{i=1}^{N}E_{i,s}\right)|\leq \xi_{2}$

10: n = n+1;

11: calculate $\eta_{EE}^{n}$ and $f(\eta_{EE}^{n})$ by Eq (11) and Eq (13);

12: if $|f\left(\eta_{EE}^{n}\right)\leq \xi_{3}$

13: obtain $P_{s}^{*}$ from Eq (23) and η^*^ from Eq (15)

## 4 Simulation results

Matlab is used to verify the advantages of the proposed method. The parameters of this paper are set as: the channel is *N* = 1, the transmitting power of the PT is *P*_*T*_ = 0.15*w*, the transmitting power of the ST is *P*_*s*_ = 0.2*w*, the interference threshold is *P*_*I*_ = 0.15*w*, and the variance of noise is *σ*^2^ = 0.1. We assumed that all channels are Rayleigh block fading channels and the simulation experiment sets the channel gains *h*_11_, *g*_12_, *h*_*ps*_ and *h*_22_ are *h* = *θd*^−*m*^, where *θ* = 0.097, *m* = 3.6 and the *d* represents the distance of PT-PR, PT-ST, ST-SR and PT-SR respectively. The energy conversion efficiency is *φ* = 0.5, and the time slot is *T* = 1*s*. The parameter settings in our paper are set with reference to [28].

As for the computational complexity, in underlay mode, assuming the number of iterations in one round is *K*. As shown in Table 1, the proposed methold needs about (4*N* + 4)*K* multiplications and 4*NK* additions to obtain *P*_*s*_, then it needs about (*N* + 1)*K* multiplications and *NK* additions to obtain λ, and(*N* + 1)*K* multiplications and *NK* additions to obtain *υ*. The computational complexity of the exhaustive search method is about *N* multiplications and *N* additions to obtain *P*_*s*_. Therefore, the computational complexity of the proposed method is higher than the exhaustive search method.

**Table 1:** The comparison of complexity and total energy consumption of different methods.

Method	Parameter	Multiplication	Addition	Complexity	Energy consumption (*P*_*End*_ − *P*_*Start*_)
Proposed method with EH	*P*_*s*_	(4*N* + 4)*K*	4*NK*	*O*_8*NK* + 4*K*_	0.04
	λ	(*N* + 1)*K*	*NK*	*O*_2*NK* + *K*_	0.013
	*v*	(*N* + 1)*K*	*NK*	*O*_2*NK* + *K*_	0.013
Exhaustive Search method	*P*_*s*_	*N*	*N*	*O*_2*N*_	0.12
	λ	*N*	*N*	*O*_2*N*_	0.12
	*v*	*N*	*N*	*O*_2*N*_	0.12
Genetic algorithm with EH	*P*_*s*_	(6*N* + 2)*K*	6*NK*	*O*_12*NK* + 2*K*_	0.095
	λ	(2*N* + 2)*K*	3*NK*	*O*_5*NK* + 2*K*_	0.036
	*v*	(2*N* + 2)*K*	3*NK*	*O*_5*NK* + 2*K*_	0.036

**Fig 3:**
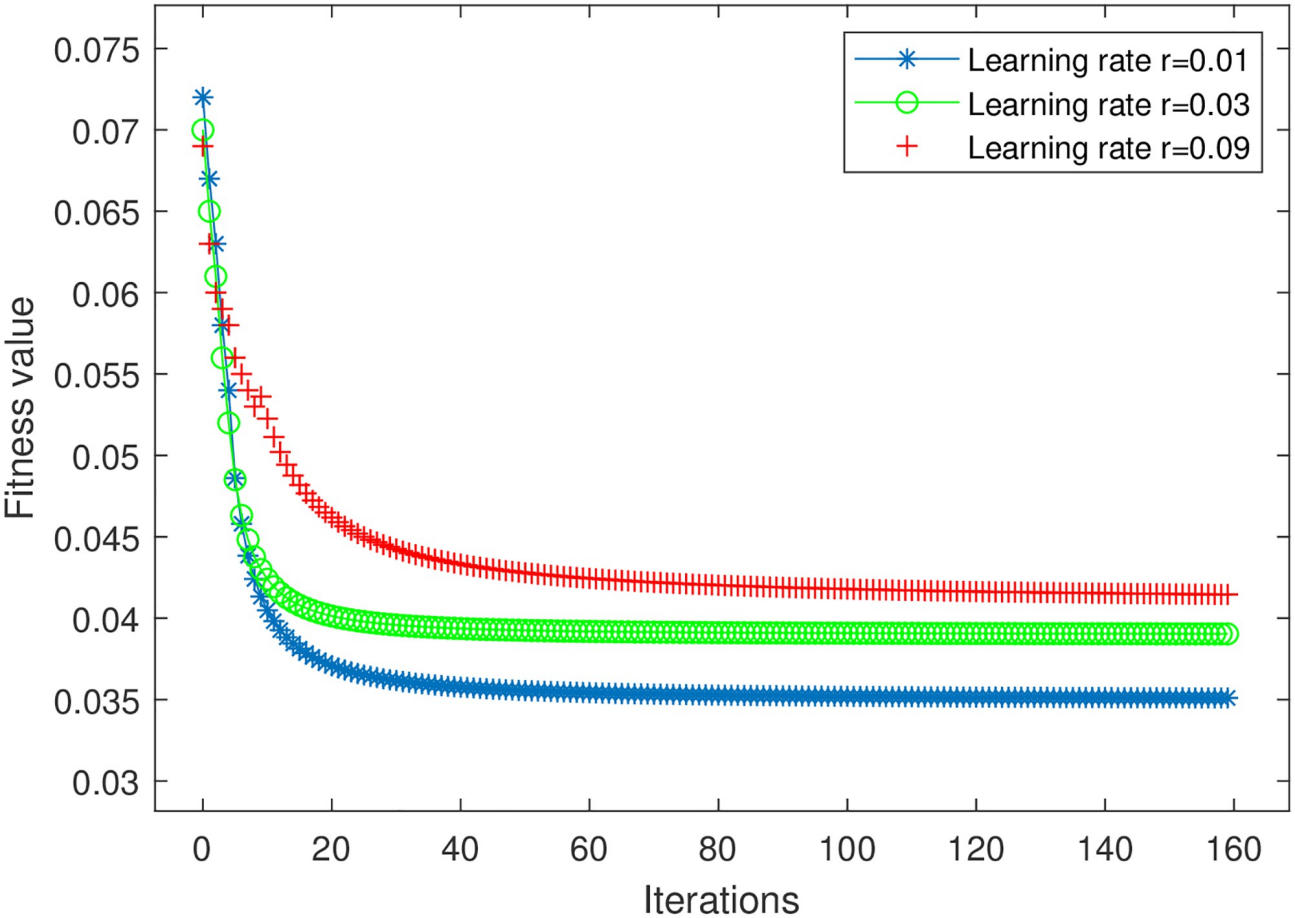
The curves of fitness value versus the number of iterations.

**Fig 4:**
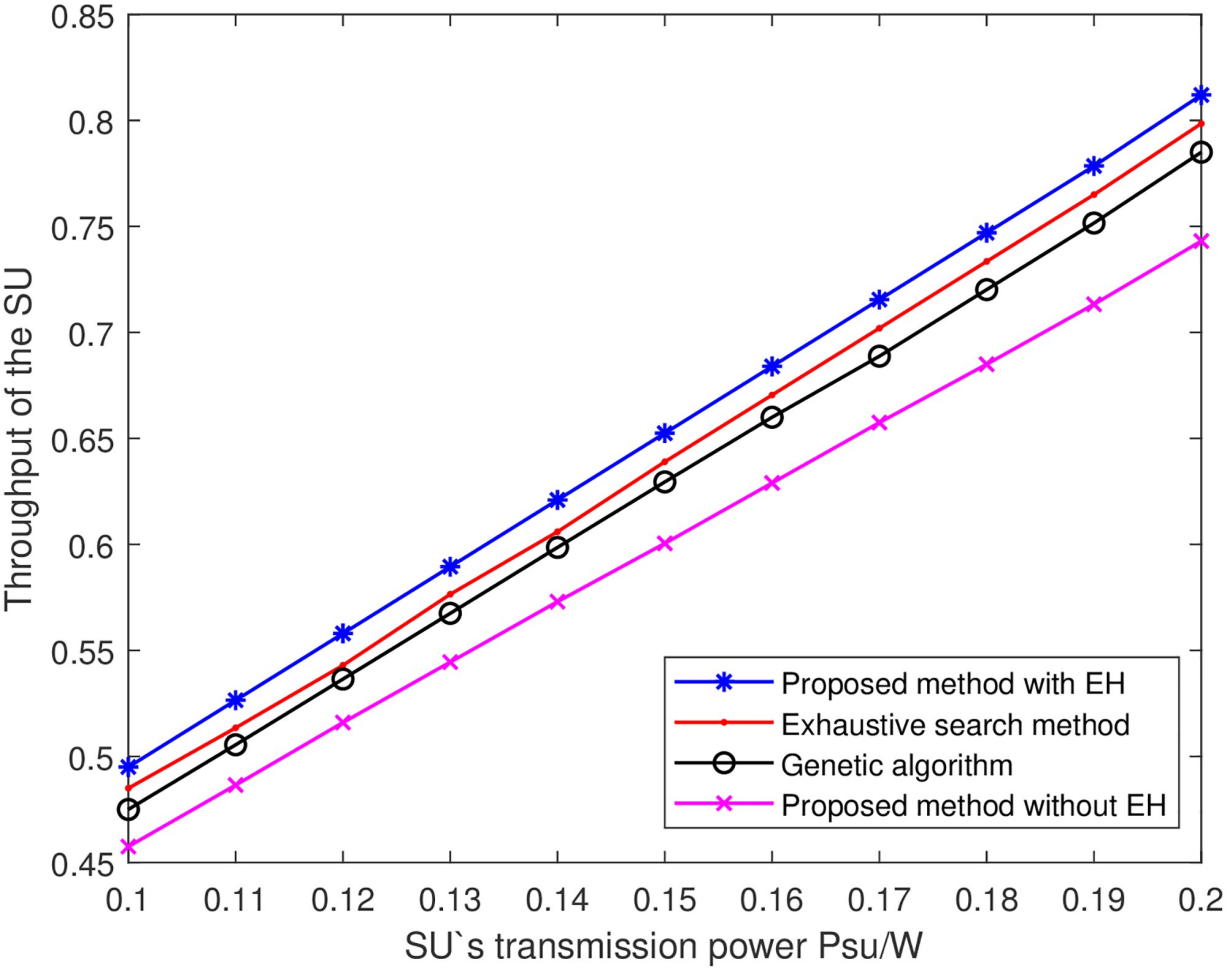
The curves of throughput of the SU versus transmitting power of the ST.

Fig 3 shows the curves of the proposed iterative method regarding the number of iterations versus the fitness value. From the Fig 3, it can be seen that the proposed iterative method gradually converges with the increase of the number of iterations when the learning rate *r* = 0.01. When the learning rate is 0.03 and 0.09, the curve is convergent, but the point is an extreme point that is not the optimal point. And the greater the learning rate, the slower the convergence curve, and even it cannot achieve convergence. Fig 4 shows the curves of cognitive user transmission power versus cognitive user throughput. Comparing the proposed iterative method with the exhaustive search method and the genetic algorithm, the proposed iterative method has better performance advantages. It can be seen from Fig 4 that the complexity of the method in this paper is higher than that of the exhaustive search method, which explains the reason for the high energy consumption of this method. But this method adopts EH technology to reduce the difference between initial energy and final energy, which makes the energy efficiency higher than exhaustive search method. At the same time, this method is a non-ergodic algorithm. Therefore, compared with the genetic algorithm using EH technology, the algorithm has lower complexity and is more suitable for energy efficiency requirements in the IoT scenario.

**Fig 5:**
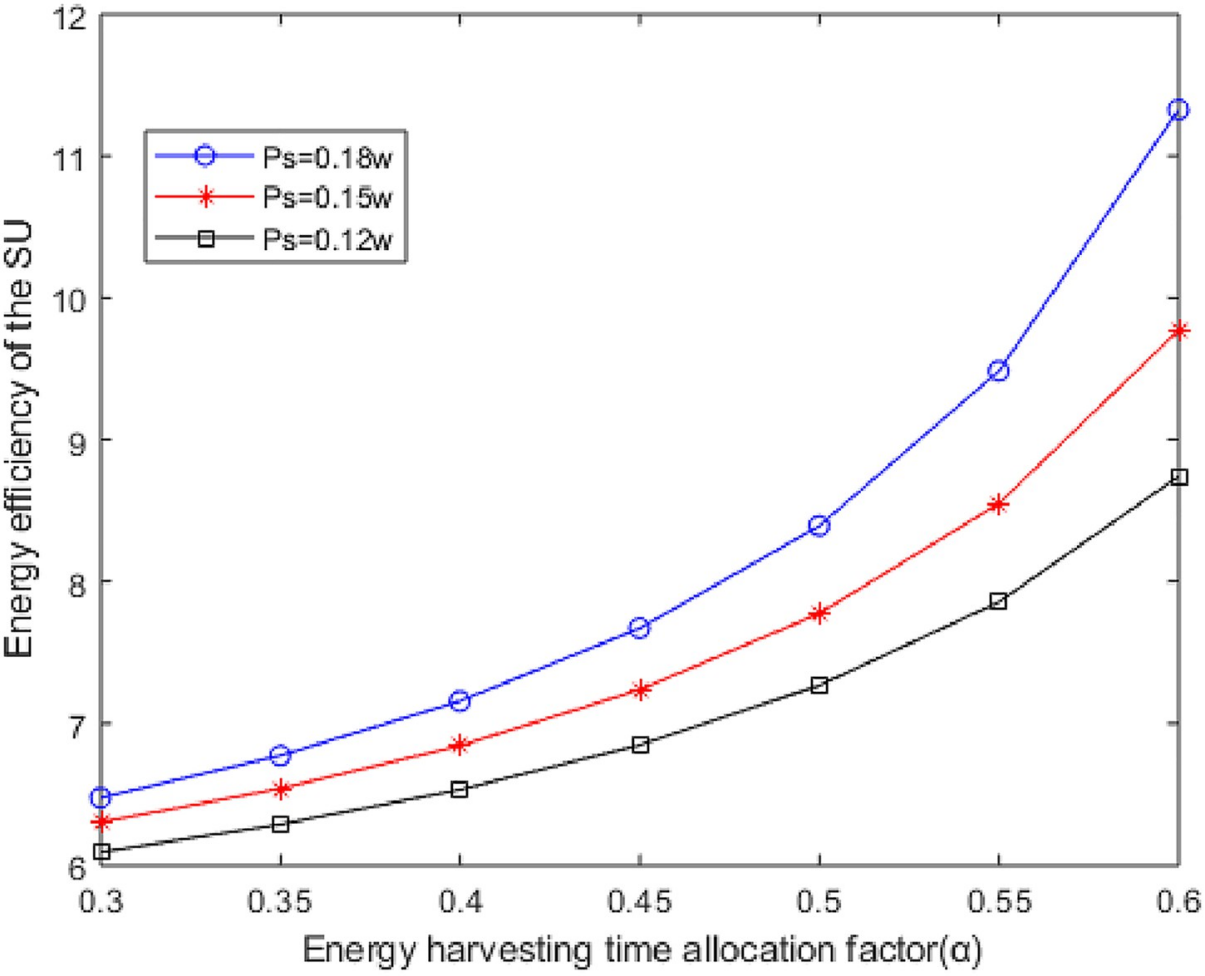
The curves of EE of the SU versus EH time allocation factor with different *P*_*s*_.

**Fig 6:**
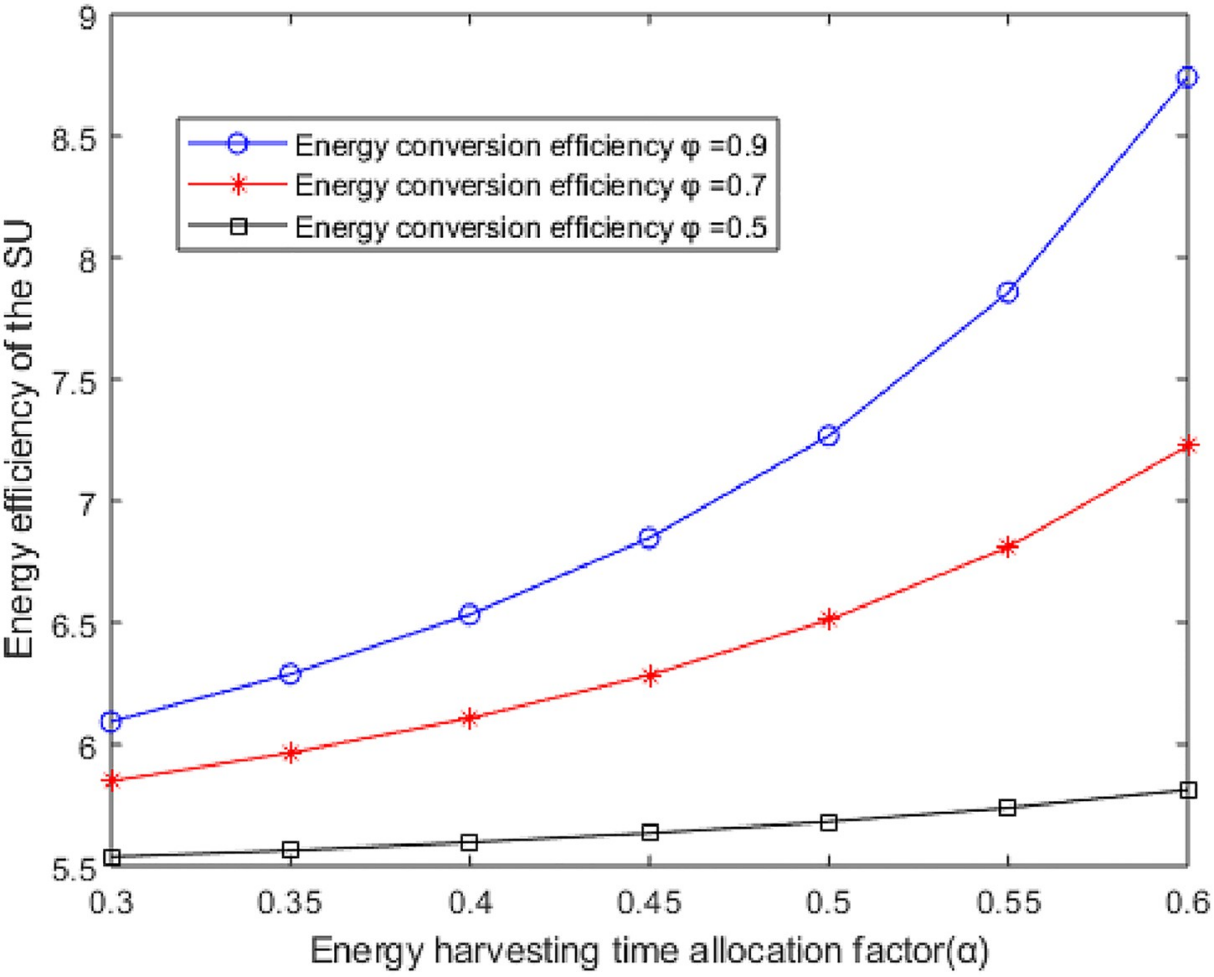
The curves of the EE of the SU versus the EH time allocation factor *α* with different *φ*.

Fig 5 shows the curves of the EE versus the EH time allocation factor at different the SU transmitting power. It can be seen from Fig 5 that the SU’s EE increases with the increase of the EH time allocation factor *α*, because the larger of the EH time allocation factor *α*, the more energy is harvested and the less energy is consumed by the non-rechargeable battery. Therefore, the SU can achieve greater the EE. In addition, when the EH time *α* is constant, the EE of the SU increases with the increase of the transmitting power. According to the analysis of Figs 5 and 6, when *α* = 0.4, the data transmission time is longer, more throughput is obtained, add the net energy consumption of SU’s is lower, so the energy efficiency of SU’s can be significantly improved. This is because the increase of the transmitting power leads to the increase of the energy ratio between the SU’s throughput and transmitting power consumption, which improves the EE of the system. Fig 4 shows that the rate of growth of the SUs’ EE has been a gradual increase in *α*, and that as the transmitted power goes up, so will the EE at the same *α*.

Fig 4 depicts the curves of the EH time allocation factor versus the EE at different energy conversion efficiency *φ*. It can be seen that the EE of the SU increases with the increase of the EH time allocation factor with the same reason as Fig 5. When the EH time allocation factor is constant, the EE of the SU increases with the increase of energy conversion efficiency *φ*. As the increase of energy conversion efficiency *φ* leads to the increase of energy collected at the ST, the energy consumed by the non-rechargeable battery is reduced and the system the EE is improved.

**Fig 7:**
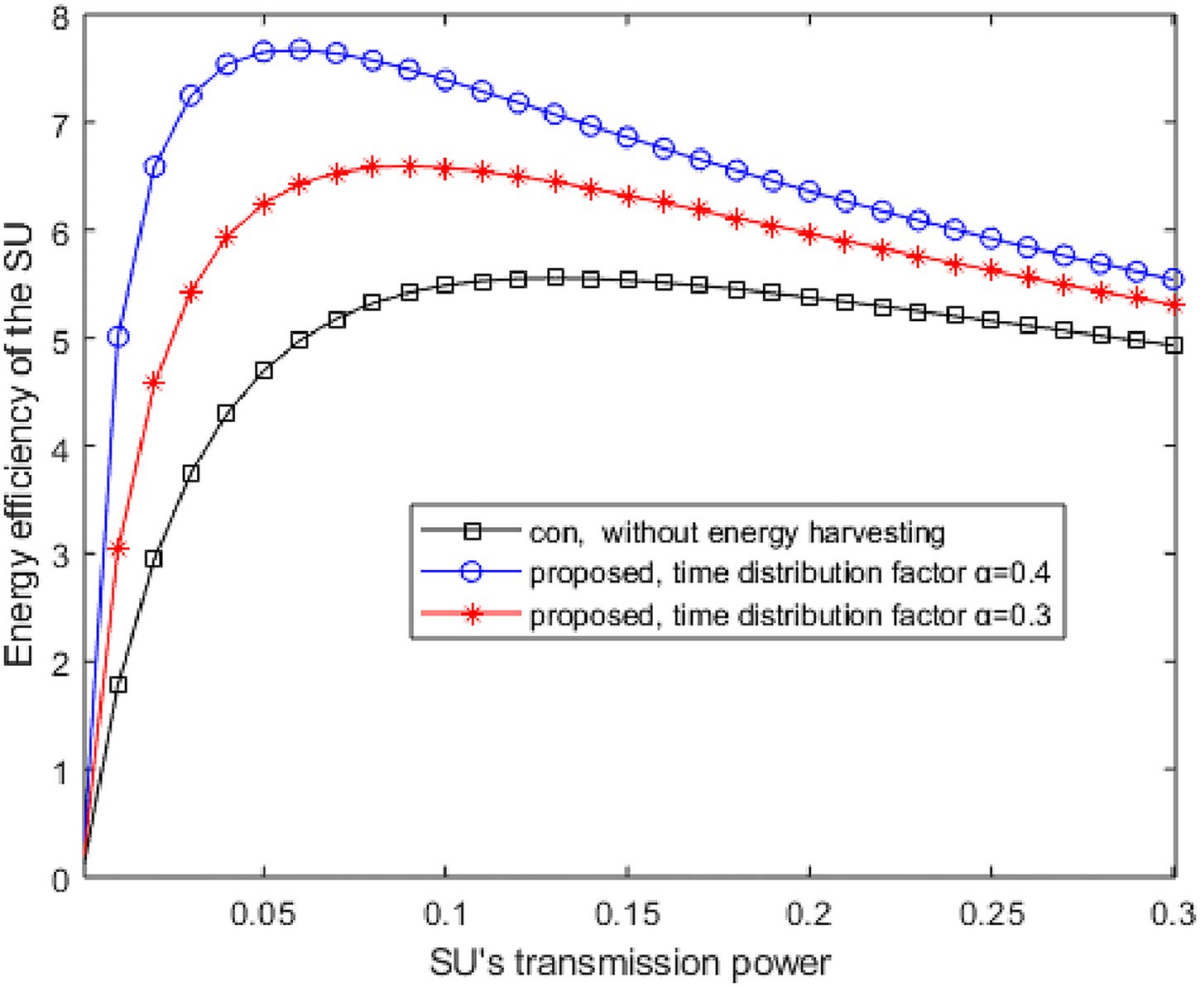
The curves of the EE with and without EH versus transmitting power of the ST.

Fig 7 illustrates the curves of the EE with and without EH versus transmitting power of the ST. In Fig 7, the EE increases at the beginning and then decreases gradually with the increase of the ST’s transmitting power. As the transmission power of the SU increases gradually, the increase rate of throughput is larger than the network energy consumption in the early stage and smaller than the network energy consumption in the later stage. The EE is related to the throughput of the SU and the energy consumption of the network, therefore, there exists an optimal transmitting power value of the ST to maximize the EE of the SU. [5] proposed an underlay cognitive wireless network where a secondary transmission link coexists with one primary transmission link. This work does not consider EH. Under the constraints of transmitting power and Interference power, the EE problem is transformed into a convex optimization problem by Dinkelbach’s method. The Lagrange multiplier method and KKT conditions are used to optimize the transmission power of the SU to obtain the optimal power allocation strategy. Fig 7 shows that the average energy efficiency of the proposed method at *α* = 0.4 and *α* = 0.3 is higher than that of the comparison method and that less transmission power is needed for the proposed method to reach its most energy efficient point than for the comparison method. The simulation results show that the EE of the proposed method is obviously higher than that of the reference [5] method.

**Fig 8:**
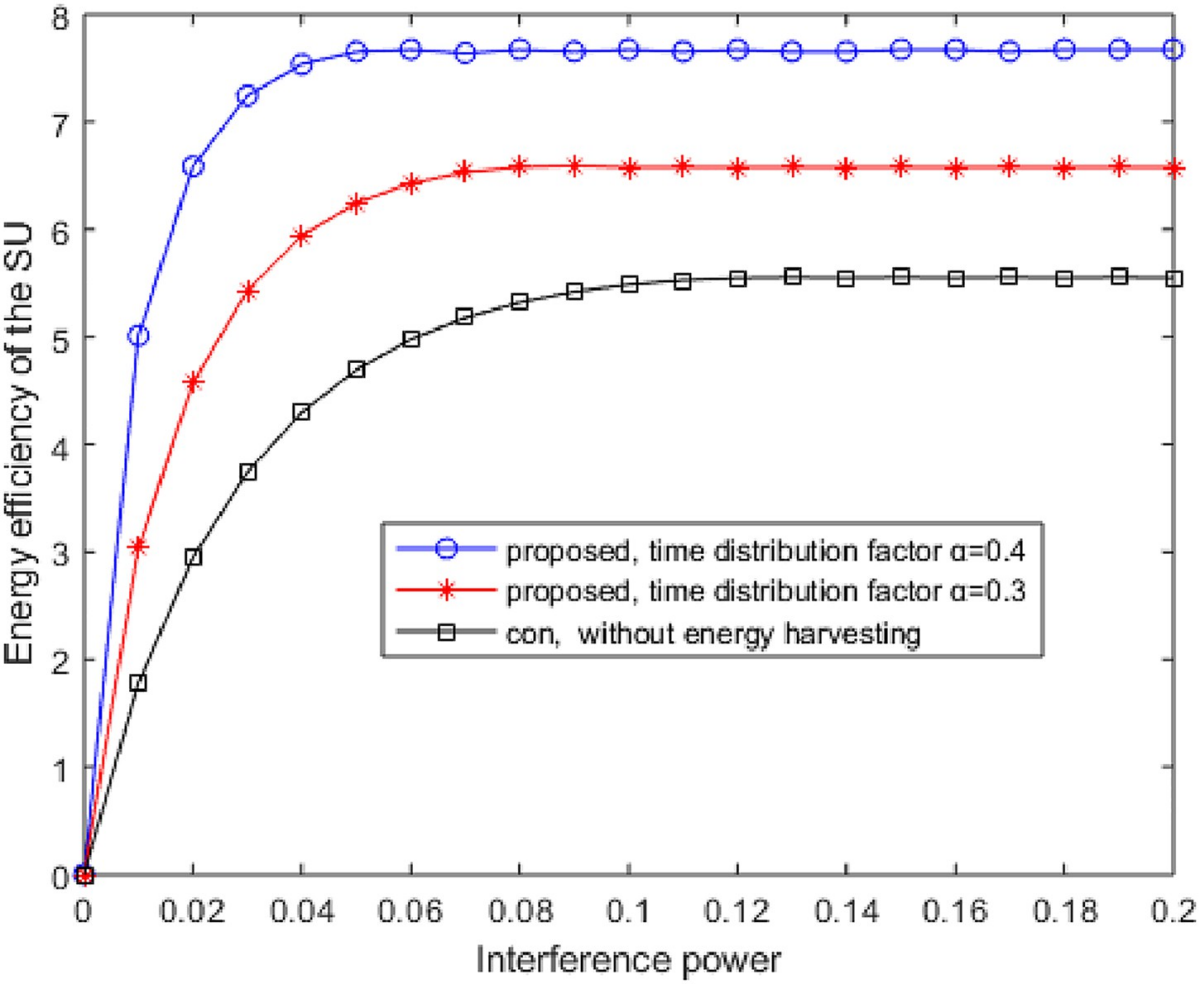
The curves of the EE with and without EH versus the interference power *P*_*I*_.

Fig 8 shows the curves of the EE with and without EH versus Interference power *P*_*I*_. As the Interference power increases, the EE increases at the beginning and then gradually converges. It can be seen that when the Interference power *P*_*I*_ starts to increase, the EE also increases. When it reaches a certain value, the EE reaches the maximum. Although the Interference power increases further, the EE maximization of the system remains in a stable range. Simulation shows that the EE of the proposed method for the SU is significantly greater than the conventional method in reference [5].

## 5 Conclusion

The EE maximization problem of EH based underlay cognitive wireless networks is modeled and solved in this paper. The SU stores the harvested energy in a limited capacity of rechargeable batteries to reduce energy consumption on the non-rechargeable battery. The objective function of maximizing the EE is derived under the interference power and energy constraints. Optimization of EH constraints to select the optimal time allocation factor. The energy-efficient optimal power allocation method is proposed by fractional programming and Lagrange multiplier, which is achieved to maximize the EE of SU. The simulation results show that compared with the conventional EE optimization method, the proposed method has more performance advantages and the EE of the system has been improved by about 15%-25%.
