# Correction: Optimal resource allocation method for energy harvesting based underlay Cognitive Radio networks

**DOI:** 10.1371/journal.pone.0315212

**Published:** 2024-12-05

**Authors:** Jianbin Liao, Hongliang Yu, Weibin Jiang, Ruiquan Lin, Jun Wang

There are errors in the author affiliations. The correct affiliations are as follows:

Jianbin Liao^1,2,3^, Hongliang Yu^1,3^, Weibin Jiang^4^, Ruiquan Lin^4^, Jun Wang^4^

**1** College of Marine Engineering, Jimei University, Xiamen, China, **2** School of Marine Engineering, Dalian Maritime University, Dalian, China **3** Fujian Province Key Laboratory of Ship and Ocean Engineering, Xiamen, Fujian, China, **4** College of Electrical Engineering and Automation, Fuzhou University, Fuzhou, China.
